# Sulforaphane inhibits thyroid cancer cell growth and invasiveness through the reactive oxygen species-dependent pathway

**DOI:** 10.18632/oncotarget.4542

**Published:** 2015-07-17

**Authors:** Liping Wang, Zhufang Tian, Qi Yang, Heng Li, Haixia Guan, Bingyin Shi, Peng Hou, Meiju Ji

**Affiliations:** ^1^ Department of Endocrinology, The First Affiliated Hospital of Xi'an Jiaotong University School of Medicine, Xi'an 710061, P.R. China; ^2^ Department of Endocrinology, Xi'an Central Hospital, Xi'an 710003, P.R. China; ^3^ Department of Endocrinology and Metabolism, The First Affiliated Hospital of China Medical University, Shenyang 110001, P.R. China; ^4^ Center for Translational Medicine, The First Affiliated Hospital of Xi'an Jiaotong University School of Medicine, Xi'an 710061, P.R. China

**Keywords:** thyroid cancer, sulforaphane (SFN), reactive oxygen species (ROS), signaling pathways

## Abstract

Sulforaphane (SFN), a natural compound derived from broccoli/broccoli sprouts, has been demonstrated to be used as an antitumor agent in different types of cancers. However, its antitumor effect in thyroid cancer remains largely unknown. The aim of the study was to investigate the therapeutic potential of SFN for thyroid cancer and explore the mechanisms underlying antitumor effects of SFN by *in vitro* and *in vivo* studies. Our data demonstrated that SFN significantly inhibited thyroid cancer cell proliferation in a dose- and time-dependent manner, induced G2/M phase cell cycle arrest and apoptosis, and inhibited thyroid cancer cell migration and invasion by suppressing epithelial-mesenchymal transition (EMT) process and expression of *Slug, Twist, MMP-2* and *-9*. Mechanically, SFN inhibited thyroid cancer cell growth and invasiveness through repressing phosphorylation of Akt, enhancing *p21* expression by the activation of Erk and p38 signaling cascades, and promoting mitochondrial-mediated apoptosis via reactive oxygen species (ROS)-dependent pathway. Growth of xenograft tumors derived from thyroid cancer cell line FTC133 in nude mice was also significantly inhibited by SFN. Importantly, we did not find significant effect of SFN on body weight and liver function of mice. Collectively, we for the first time demonstrate that SFN is a potentially effective antitumor agent for thyroid cancer.

## INTRODUCTION

Thyroid cancer is a common endocrine malignancy that has rapidly increased in global incidence in the past 15 years [1, 2]. It is now the fifth most common cancer in women [2]. Primary thyroid cancer is classified into three major histopathologic types [3]: differentiated thyroid cancers (DTCs), including papillary (PTC, 85% of cases) and follicular (FTC, 5%–10%); medullary thyroid cancer (MTC, 5%); anaplastic thyroid cancer (ATC, 1%). Although the death rate of thyroid cancer is relatively low and general prognosis is excellent, 10%–20% of patients with DTC can develop into more aggressive and *dedifferentiated* forms of *thyroid cancer* either at the initial presentation or as a recurrence, which is closely correlated with patient mortality [3, 4].

Conventional surgical thyroidectomy with adjuvant ablation by radioiodine treatment has been the mainstay of thyroid cancer treatment, however, about half of the patients with advanced disease will not respond adequately to such therapy [5]. Recent advances in understanding the molecular pathogenesis of thyroid cancer have shown great promise to develop more effective treatment for thyroid cancer [3]. This has mainly resulted from the identification of molecular alterations in major signaling pathways, such as the RAS/RAF/MEK/MAPK/ERK (MAPK) and PI3K/Akt pathways, which play critical roles in cell transformation, survival and metastasis, and therefore become classical therapeutical targets for thyroid cancer [3, 5, 6]. In addition to targeted therapies, in recent years, some of natural product-derived drugs also display potent antitumor activity in thyroid cancer, such as paclitaxel, vincristine, vinorelbine and shikonin [7–10].

Sulforaphane (SFN) is a naturally occurring isothiocyanate derived from cruciferous vegetables, especially broccoli. It has been proved to be an important candidate cancer preventive agent that has high activity in diverse cancers, including colon cancer [11], bladder cancer [12], prostate cancer [13, 14], breast cancer [15] and leukemia [16, 17]. However, its antitumor effect in thyroid cancer remains largely unknown. In this study, we used a panel of authenticated thyroid cancer cell lines and primary thyroid cancer cells to test *in vitro* and *in vivo* therapeutic potential of SFN and attempted to explore its antitumor mechanisms in thyroid cancer.

## RESULTS

### SFN inhibits thyroid cancer cell proliferation

**Figure 1:**
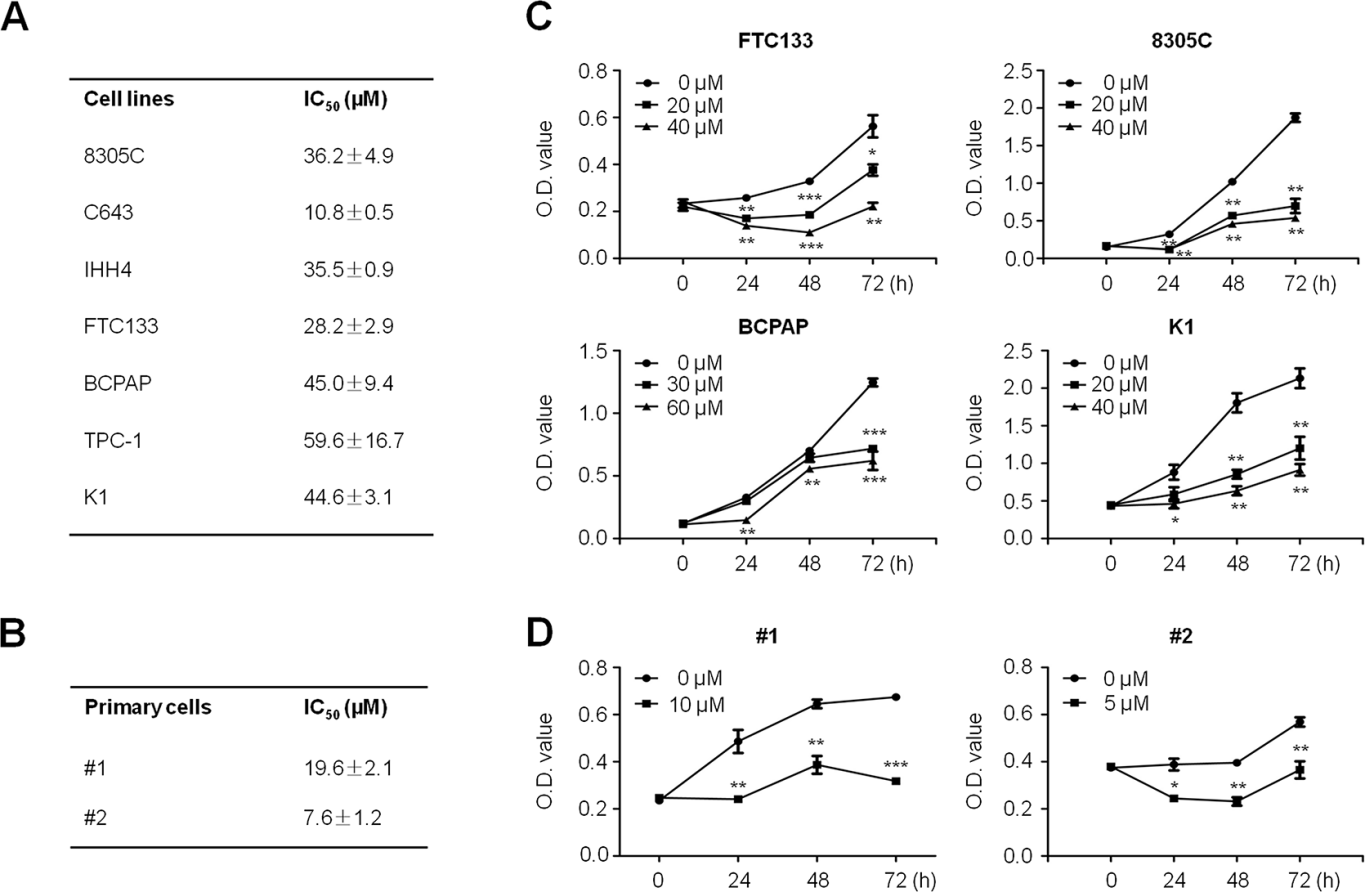
Proliferation-inhibitory of thyroid cancer cell lines and primary thyroid cancer cells by SFN Thyroid cancer cell lines **A.** and primary cancer cells **B.** were treated with different doses of SFN for 48 h. MTT assay was performed to evaluate cell growth ability and IC_50_ values were calculated using the Reed-Muench method (see Supplementary data). Data were presented as mean ± SD. Time course of cell proliferation was measured by MTT assay in each cell line **C.** and primary cancer cells **D.** treated with the indicated concentrations of SFN or vehicle control at the indicated time point. *, *P* < 0.05; **, *P* < 0.01; ***, *P* < 0.001.

MTT assay was performed to examine the dose and time course of the effect of SFN on cell proliferation in a panel of thyroid cell lines and primary thyroid cancer cells that were obtained from two different PTC patients. As shown in Figure 1A, we found that SFN significantly inhibited cell proliferation in thyroid cancer cell lines in a dose-dependent manner, with IC_50_ values ranging from 10.8 to 59.6 μM. We attempted to explore the association of cellular response to SFN with molecular alterations in the major components of MAPK and PI3K/Akt pathways and p53 status. However, we did not find any relationship (data not shown). In addition, our data demonstrated that primary cancer cells were also sensitive to SFN, and IC_50_ values were 7.6 μM and 19.6 μM, respectively (Figure 1B). Next, we analyzed time-dependent response of thyroid cancer cell lines and primary cancer cells to SFN. As shown in Figure 1C, SFN significantly inhibited proliferation of FTC133, 8305C, BCPAP and K1 cells at the indicated concentrations and time points. Similarly, SFN also significantly inhibited proliferation of primary cancer cells at the indicated concentrations and time points (Figure 1D).

### SFN induces cell cycle arrest and apoptosis in thyroid cancer cells

**Figure 2:**
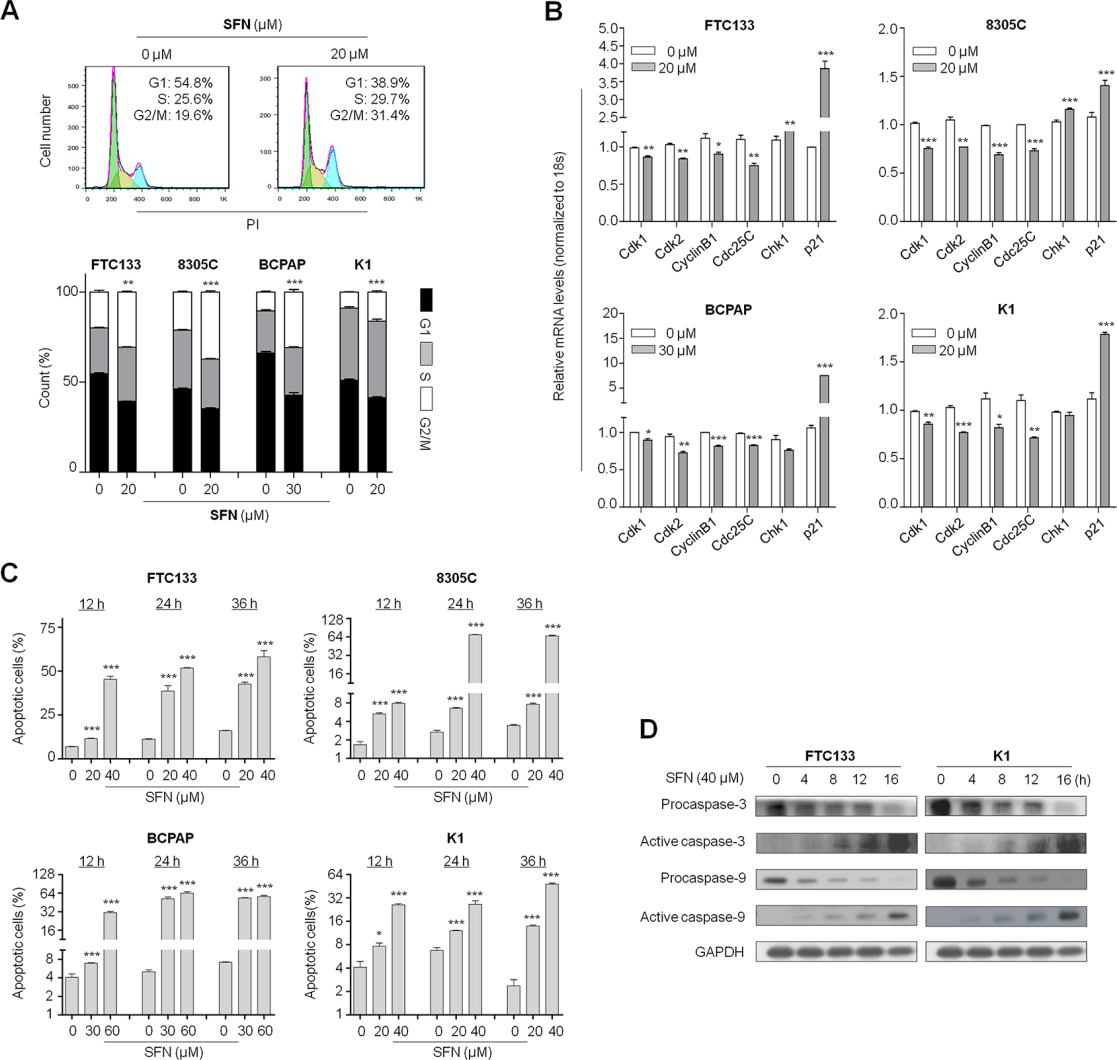
Induction of cell cycle arrest and apoptosis by SFN in thyroid cancer cells **A.** Thyroid cancer cells were treated with the indicated concentrations of SFN or vehicle control for 24 h. DNA content was then measured by flow cytometry to determine cell cycle fractions. Representative flow cytometric histograms of FTC133 cells treated with vehicle control or 20 μM SFN from three independent experiments are shown in upper panel. The percentage of FTC133, 8305C, BCPAP and K1 cells in each cell cycle phase is indicated in lower graph. **B.** Total RNA was extracted from cells treated with vehicle control or the indicated concentrations of SFN for 24 h. RT-qPCR was preformed to quantify expression of the indicated genes with *18S* rRNA as endogenous control. Data were presented as mean ± SD. *, *P* < 0.05**; *P* < 0.01; ***, *P* < 0.001. **C.** Cell apoptosis was measured by flow cytometry analysis of Annexin V-FITC/PI double-labeled cells treated with vehicle control or the indicated doses of SFN for the indicated time point. The percentage of apoptotic cells for FTC133, 8305C, BCPAP and K1 cells is indicated in bar graph. *, *P* < 0.05**; *P* < 0.01; ***, *P* < 0.001. **D.** Cells were treated with vehicle control or SFN at the indicated doses for 24 h. Cell lysates were collected and subjected to Western blotting. The antibodies against procaspase-3 and -9 and their active forms were used to determine the effect of SFN on intrinsic apoptosis related to mitochondrial dysfunction. GAPDH was used as loading control.

Given that growth inhibitory of cancer cell is usually associated with cell cycle arrest, we thus examined the effect of SFN on cell cycle in thyroid cancer cells. As shown in Figure 2A, as compared with controls, cell cycle was arrested at the G2/M phase when FTC133, 8305C, BCPAP and K1 cells were treated with the indicated doses of SFN for 24 h. The percentage of G2/M phase was increased from 19.9 ± 1.7% to 30.7 ± 0.7% in FTC133 cells, from 21.3 ± 0.8% to 37.3 ± 1.3% in 8305C cells, from 10.5 ± 0.7% to 30.9 ± 2.4% in BCPAP cells and from 8.9 ± 0.2% to 16.2 ± 1.2% in K1 cells, respectively (Figure 2A, lower panel). To explore the mechanism underlying SFN-mediated G2/M arrest, we investigated the effect of SFN on the expression of cell cycle-related genes in these four cell lines, including *Cdk1, Cdk2, CyclinB1, Cdc25C, Chk1* and *p21*. As shown in Figure 2B, SFN treatment at the indicated concentrations caused a decreased expression of *Cdk1, Cdk2, CyclinB1* and *Cdc25C* in all cell lines, whereas *Chk1* expression in FTC133 and 8305c cells and *p21* expression in all cell lines were increased by SFN treatment.

Next, we tested the effect of SFN on thyroid cancer cell apoptosis. As shown in Figure 2C, FTC133, 8305C, BCPAP and K1 cells treated with the indicated concentrations of SFN at the indicated time point showed a dramatic increase in both early and late apoptosis as compared with controls, and this effect was dose-dependent. Induction of apoptosis was further confirmed by detecting the expression of apoptosis-related proteins in FTC133 and K1 cells. As shown in Figure 2D, 40 μM SFN enhanced the protein levels of the active form of caspase-3 and -9, and decreased the protein levels of procaspase-3 and -9 in a time-dependent manner in these two cell lines.

### SFN induces production of reactive oxygen species (ROS) and the loss of mitochondrial membrane potential (MMP) in thyroid cancer cells

**Figure 3:**
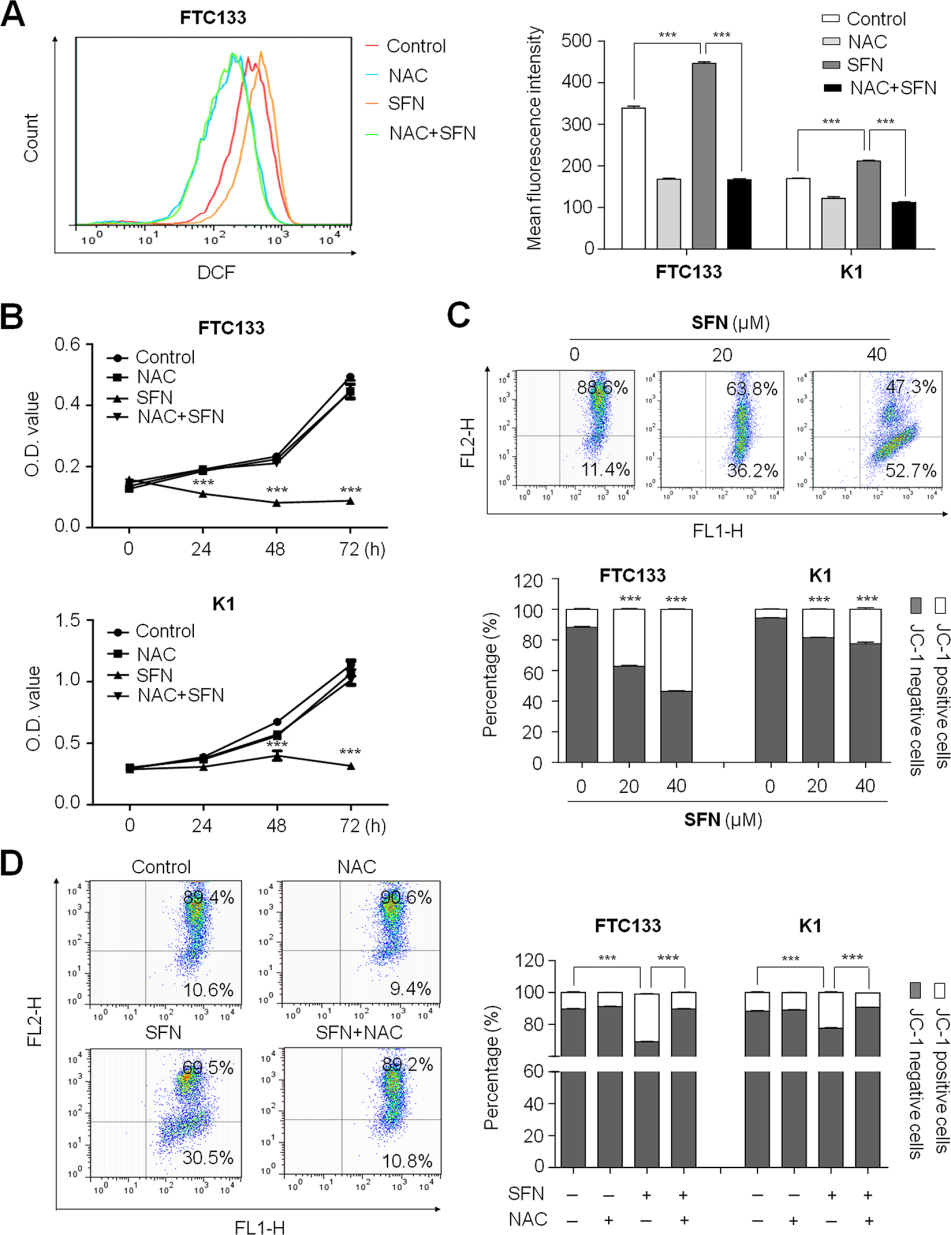
Induction of ROS production and the loss of mitochondrial membrane potential (MMP) by SFN in thyroid cancer cells **A.** FTC133 and K1 cells were treated with 40 μM SFN and 20 mM NAC each alone or in combination for 4 h. DCF staining was used to evaluate ROS production. The fluorescence intensity of ROS-positive cells is shown as indicated in FTC133 cells (left panel). The right bar graphs show mean ± SD of the values from three independent experiments in FTC133 and K1 cells. ***, *P* < 0.001. **B.** Time course of cell proliferation was measured by MTT assay in FTC133 and K1 cells treated with 40 μM SFN and 20 mM NAC each alone or in combination. ***, *P* < 0.001. **C.** FTC133 and K1 cells treated with the indicated doses of SFN for 12 h. JC-1 staining and flow cytometry were performed to evaluate the effect of SFN on MMP. Dot plots for JC-1-stained FTC133 cells are shown in upper panel. The right lower region represents JC-1 positive cells (or apoptotic cells). The right upper region represents JC-1 negative cells (or non-apoptotic cells). The percentage of JC-1 positive and JC-1 negative cells for FTC133 and K1 cells is indicated in lower panel. ***, *P* < 0.001. **D.** FTC133 and K1 cells were treated with 40 μM SFN and 20 mM NAC each alone or in combination for 4 h. Flow cytometry profiles for JC-1 stained FTC133 cells are shown in left panel. The percentage of JC-1 positive and JC-1 negative cells for FTC133 and K1 cells are indicated in right panel. ***, *P* < 0.001.

It is well documented that cell death induced by some antitumor agents is associated with ROS generation and the perturbation of mitochondrial functions, including SFN [14, 15]. Thus, we performed DCF staining assay to analyze ROS production induced by SFN in this study. As shown in Figure 3A, as compared with controls, ROS was significantly induced in both FTC133 and K1 cells when cells were treated with 40 μM SFN for 4 h. The mean fluorescence intensity was increased from 339.0 ± 2.7 to 446.7 ± 3.5 (*P* < 0.001) in FTC133 cells and from 169.7 ± 1.5 to 212.0 ± 1.7 (*P* < 0.001) in K1 cells. Moreover, we further measured ROS levels in FTC133 and K1 cells treated with SFN for 12 h, 24 h and 48 h, respectively. The result showed that ROS production reached to the peak at 24 h in FTC133 cells and at 12 h in K1 cells, respectively, and then decreased continuously as compared to the controls (Supplementary Figure S1). Next, ROS scavenger, N-acetylcystein (NAC), was used to explore the role of ROS-induced by SFN in growth inhibitory of thyroid cancer cells. As expected, 20 mM NAC dramatically decreased SFN-induced ROS production (Figure 3A), and significantly blocked cell cycle arrest and apoptosis induced by SFN in both FTC133 and K1 cells (Supplementary Figure S2). As a result, anti-proliferative effect of SFN was *almost completely* abolished by NAC in these two cells (Figure 3B). To further determine the role of ROS in SFN-mediated growth inhibitory, we selected another ROS scavenger, ascorbate (ASC), to treat FTC133 and K1 cells. Like NAC, ASC treatment significantly attenuated anti-proliferative effect of SFN in these two cell lines (Supplementary Figure S3).

Given that loss of MMP is a hallmark of apoptosis, representing an early event coinciding with caspase activation [18], in this study, mitochondrial probe *5,5*′*, 6,6*′-*Tetrachloro-1,1*′*,3,3*′-*Tetraethylbenzimidazolyl*- *Carbocyanine* iodide (JC-1) was used to test the effect of SFN on MMP in FTC133 and K1 cells. In non-apoptotic cells, JC-1 exists as dimer and accumulates as aggregate in the mitochondria, which appears red. Whereas, in apoptotic and necrotic cells, JC-1 exists in a monomeric form and stains the cytosol green. Typical FL-1/FL-2 dot plots for JC-1-stained FTC133 cells were exhibited in Figure 3C (upper panels). The green fluorescing monomers shown in the lower region represented apoptotic cells. As shown in Figure 3C (lower panel), cells exposed to SFN at the indicated concentrations exhibited a dose-dependent decrease in JC-1 staining as compared to control cells, indicating loss of MMP in SFN-treated cells. Similarly, we also found that the effect of SFN on MMP could be almost completely reversed by NAC (Figure 3D). Collectively, loss of MMP induced by ROS may act as an important mediator of SFN-induced cell death in thyroid cancer cells.

### SFN inhibits thyroid cancer cell migration and invasion

**Figure 4:**
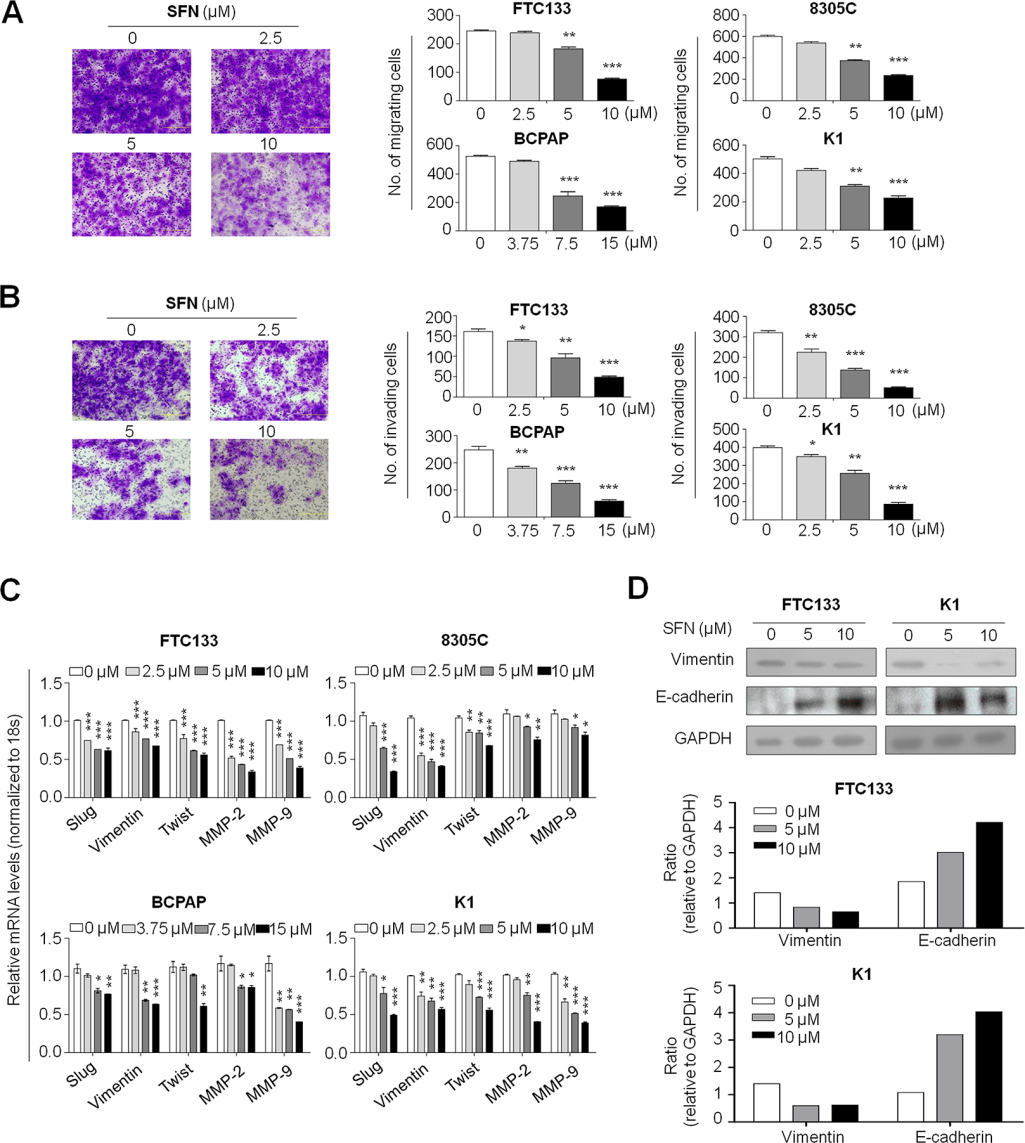
Inhibition of thyroid cancer cell migration and invasion by SFN Cells were treated with vehicle control or the indicated concentrations of SFN for 24 h. Transwell migration assay **A.** and Matrigel *invasion assay* **B.** were performed to test the effect of SFN on thyroid cancer cell migration and invasion, respectively. Representative images of cell migration/invasion of FTC133 cells are shown in left panel. The bar graphs on the right side show mean ± SD of the numbers of migrating/invading FTC133, 8305C, BCPAP and K1 cells from three independent experiments. *, *P* < 0.05**; *P* < 0.01; ***, *P* < 0.001. **C.** Total RNA was extracted from cells treated with vehicle control or the indicated concentrations of SFN for 24 h. RT-qPCR was then preformed to evaluate expression of metastasis-related genes including *Slug, Vimentin, Twist, MMP-2*, and *-9*. Expression levels of these genes were normalized with *18S* rRNA levels. Data were presented as mean ± SD. *, *P* < 0.05; **, *P* < 0.01; ***, *P* < 0.001. **D.** Cells were treated with SFN at the indicated concentrations for 24 h. Cell lysates were collected and subjected to Western blotting. E-cadherin and Vimentin were blotted to evaluate the effect of SFN on EMT process of thyroid cancer cells. Shown in the middle and lower panels is a quantitative illustration of levels of Vimentin and E-cadherin protein using densitometry to measure the density of the corresponding bands on western blot shown in the upper panel. GAPDH was used as loading control.

The inhibitory effect of SFN on cell migration and invasive ability was determined by the transwell assay. As showed in Figure 4A, as compared with controls, there were a significantly lower number of migrated cells in FTC133, 8305C, BCPAP and K1 cells treated SFN at the indicated concentrations, respectively. Similarly, the Matrigel assays showed that SFN at the indicated concentrations significantly inhibited the invasive potential of thyroid cancer cells (Figure 4B). Notably, inhibition of thyroid cancer cell migration and invasion by SFN was a dose-dependent manner. To clarify the related mechanisms, we tested the effect of SFN on expression of metastasis-related genes in these cell lines, including *Slug, Vimentin, Twist, MMP-2* and *-9*. As shown in Figure 4C, SFN dramatically inhibited the expression of these genes in a dose-dependent manner in all cell lines. In addition, we also investigated the effect of SFN on protein expression of epithelial-mesenchymal transition (EMT) markers E-cadherin and Vimentin in FTC133 and K1 cells. As expected, SFN at the indicated concentrations substantially promoted E-cadherin expression and reduced Vimentin expression in these two cell lines (Figure 4D). These observations suggest that the decrease in metastasis-associated phenotypes may be link to inhibition of expression of metastasis-related genes and EMT process by SFN.

**Figure 5:**
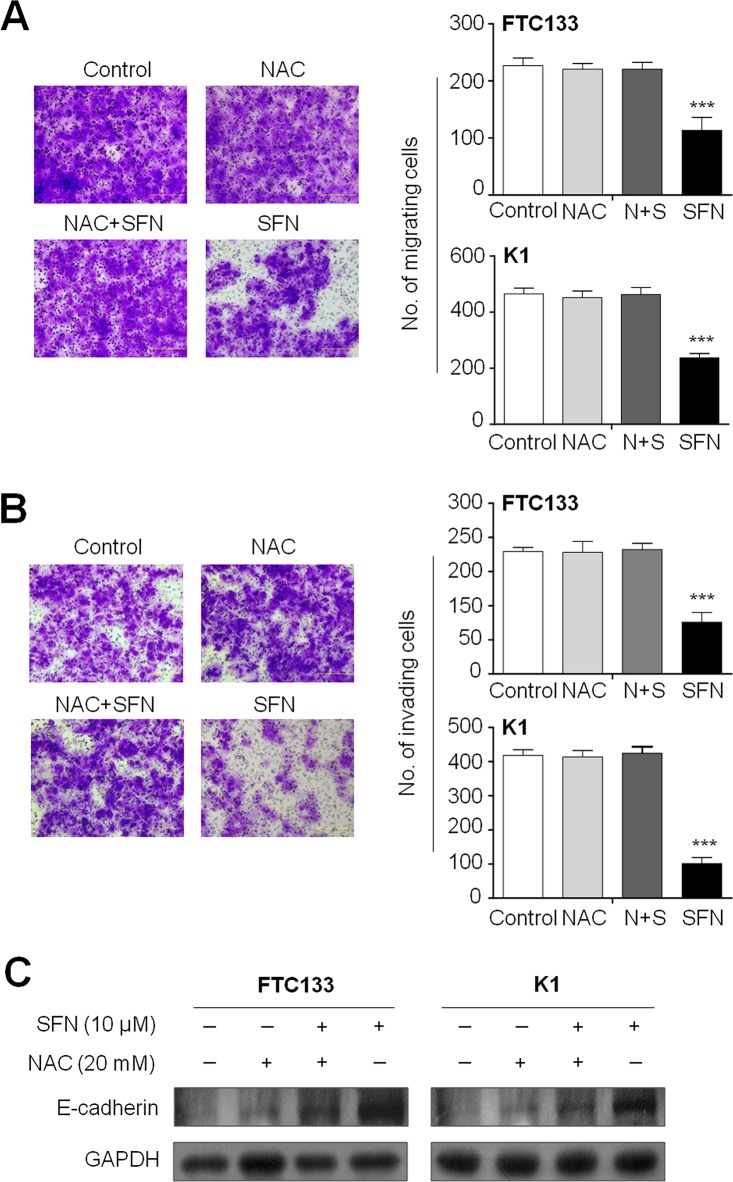
The effect of elimination of ROS by NAC on migration, invasion and E-cadherin expression Cells were treated with 10 μM SFN and 20 mM NAC each alone or in combination for 24 h. Transwell migration assay **A.** and Matrigel *invasion assay* **B.** were performed to test the effect of ROS on thyroid cancer cell migration and invasion, respectively. Representative images of cell migration/invasion of FTC133 cells are shown in left panel. The bar graphs on the right side show mean ± SD of the numbers of migrating/invading FTC133 and K1 cells from three independent experiments. N+S, NAC+SFN; ***, *P* < 0.001. **C.** Intracellular amount of E-cadherin was compared among FTC133 and K1 cells treated with 10 μM SFN and 20 mM NAC each alone or in combination for 24 h using western blot analysis.

Next, we attempted to test whether ROS was involved in thyroid cancer cell migration and invasion. As shown in Figure 5A and 5B, the inhibiting effects of SFN on migration and invasion were *completely* abolished by NAC in these two cell lines. Moreover, NAC treatment significantly decreased E-cadherin expression induced by SFN in these cell lines (Figure 5C). These findings further support the role of ROS in thyroid cancer cell invasiveness.

### SFN modulates the activities of major signaling pathways in thyroid cancer cells through a ROS-dependent mechanism

**Figure 6:**
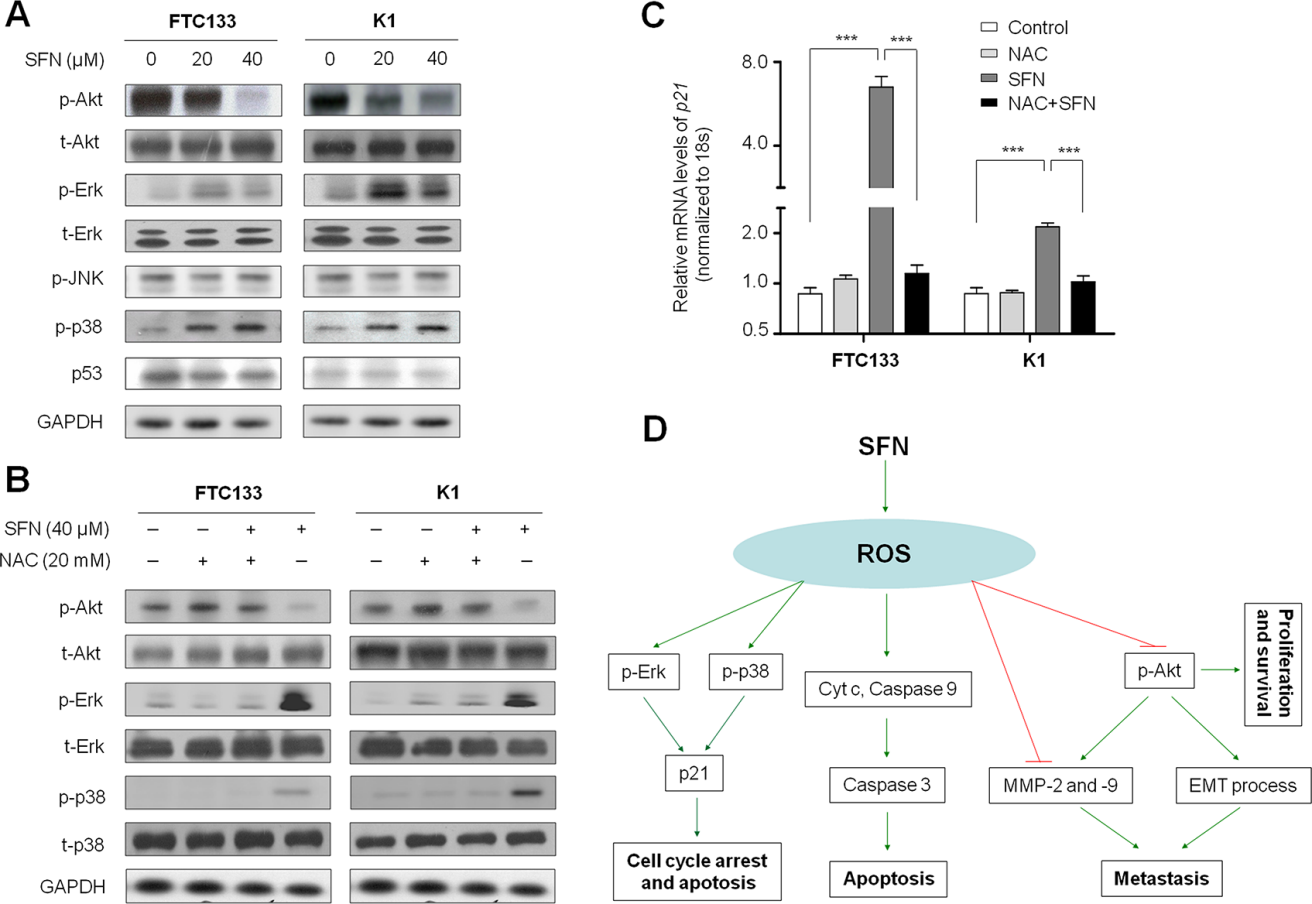
Effect of SFN on the activities of major signaling pathways in thyroid cancer cells Cells were treated with SFN at the indicated doses for 24 h. Cell lysates were collected and subjected to Western blotting. **A.** The antibodies against phospho-Akt^Ser473^ (p-Akt), total-Akt (t-Akt), phospho-Erk (p-Erk), total-Erk (t-Erk), phosphor-JNK and phosphor-p38 (p-p38) were used to determine the effect of SFN on the activities of the PI3K/Akt, MAPK/Erk, MAPK/JNK and MAPK/p38 signaling pathways. The effect of SFN on p53 accumulation was determined by blotting p53. GAPDH was used as loading control. **B.** Intracellular amount of p-Akt, total-Akt(t-Akt), p-Erk, total-Erk (t-Erk), p-p38 and total-p38 (t-p38) were compared among FTC133 and K1 cells treated with 40 μM SFN and 20 mM NAC each alone or in combination for 24 h. **C.** Total RNA was extracted from cells treated with 20 μM SFN and 20 mM NAC each alone or in combination for 24 h. RT-qPCR was preformed to quantify *p21* expression. Data were presented as mean ±SD. ***, *P* <0.001. **D.** A simplified model for the mechanisms underlying antitumor effects of SFN in thyroid cancer.

To explore the mechanisms underlying the antitumor activity of SFN, we tested the effect of SFN on the activities of PI3K/Akt, MAPK/Erk, MAPK/JNK and MAPK/p38 pathways, which play an important role in cell proliferation, metastasis and survival in thyroid cancer [3]. As shown in Figure 6A, SFN significantly inhibited phosphorylation of Akt in a dose-dependent manner in both FTC133 and K1 cells. Moreover, we found that SFN enhanced phosphorylation of Erk and p38 in these two cell lines, and promoting effect of SFN on phosphorylation of p38 was dose-dependent. However, we did not find the effect of SFN on phosphorylation of JNK (Figure 6A). Given that tumor suppressor p53 plays a central role in the regulation of cell cycle and apoptosis, we thus investigated the effect of SFN on p53 activity in these two cell lines. We did not find the effect of SFN on the accumulation of p53 protein in both FTC133 and K1 cells harboring mutant-type and wild-type p53, respectively (Figure 6A). However, our data showed that the expression of its downstream target gene *p21* was increased by SFN in thyroid cancer cells (Figure 2B), suggesting that SFN enhances *p21* expression by a p53-independent pathway. This is supported by a previous study that elevation of ROS has been demonstrated to result in p53-independent accumulation of p21 through enhancing the activity of Erk and p38 [19].

Next, we attempted to determine the role of ROS in the modulation of these signaling pathways in FTC133 and K1 cells. As expected, combination of SFN and NAC at the indicated concentrations remarkably restored phosphorylation of Akt and inhibited phosphorylation of Erk and p38 as compared with SFN treatment alone in both FTC133 and K1 cells (Figure 6B). Accordingly, our data demonstrated that NAC dramatically inhibited SFN-induced *p21* expression in these two cell lines (Figure 6C). Taken together, our data are consistent with a model (Figure 6D) in which SFN contributes to inhibition of thyroid cancer cell growth and metastasis through repressing the activity of PI3K/Akt cascade and expression of *MMP-2* and *-9*, increasing *p21* expression by activation of MAPK/Erk and MAPK/p38 signaling pathways, and promoting mitochondrial-mediated apoptosis via a ROS-dependent mechanism.

### SFN inhibits xenograft tumor growth

**Figure 7:**
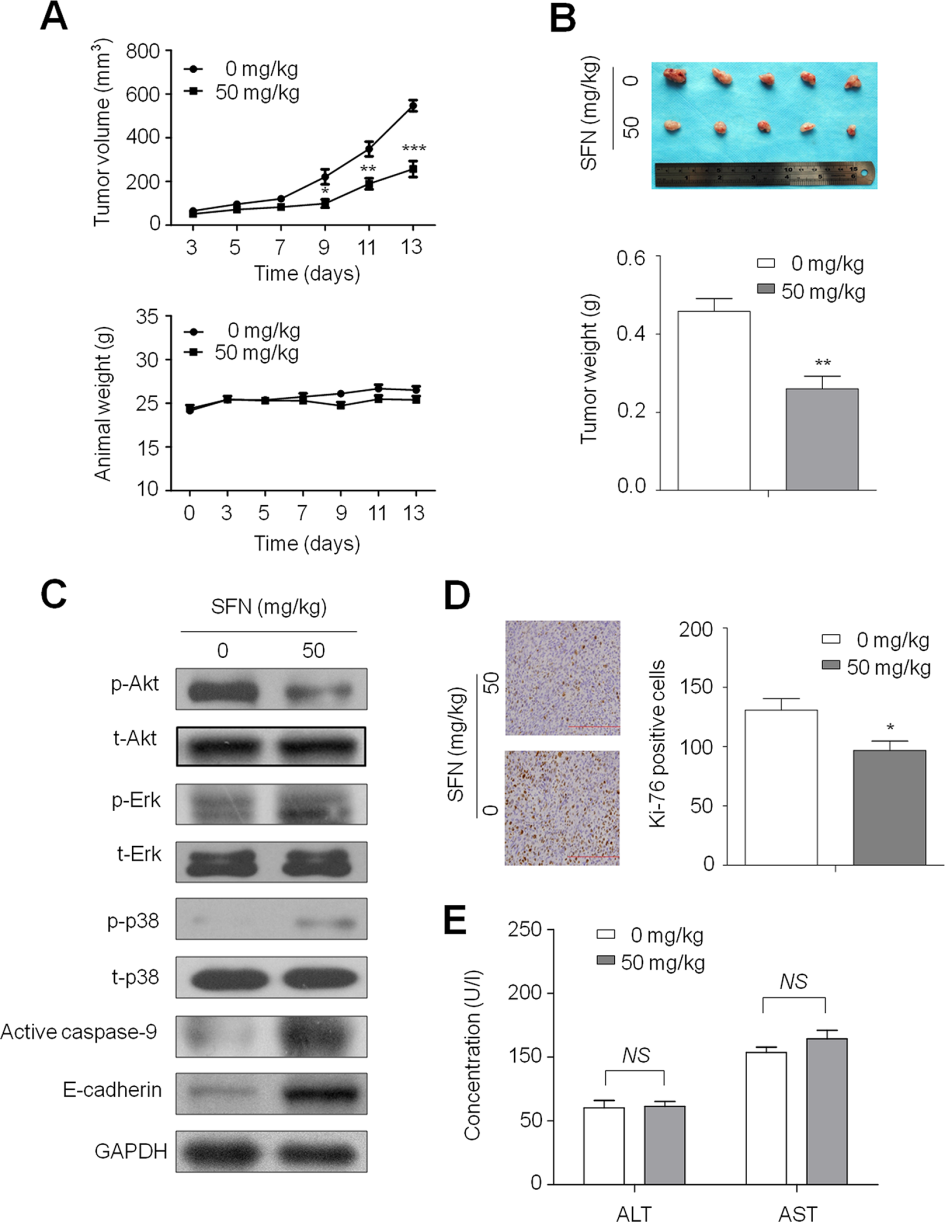
Inhibition of xenograft tumor growth by SFN **A.** Time courses of tumor growth (upper panel) and animal weight (lower panel) were measured in each group at the indicated time point of various treatments. Data were presented as mean ± SD (*n* = 5/group). *, *P* < 0.05; **, *P* < 0.01; ***, *P* < 0.001. **B.** Shown is a representative picture of tumor growth in mice treated with vehicle control and the indicated concentration of SFN (upper panel). Bar graphs represent mean of the tumor weight from control and SFN-treated mice (lower panel). Data were presented as mean ± SD (*n* = 5/group). **, *P* < 0.01. **C.** Tumors tissues from representative mice were homogenated, lysed, and then subjected to western blotting using specific primary antibodies, including p-Akt, total-Akt (t-Akt), p-Erk, total-Erk (t-Erk), p-p38, total-p38 (t-p38) and active caspase-9 and E-cadherin. GAPDH was used as loading control. **D.** Shown is representative Ki-67 staining of FTC133-derived xenograft tumors from control and SFN-treated mice (left panel). Bar graphs represent mean ± SD of the numbers of Ki-67-positive cells from 5 microscopic fields in each group (right panel). Data were presented as mean ± SD. *, *P* < 0.05. **E.** Serum activities of aspartate transaminase (AST) and alanine transaminase (ALT) were measured in control and SFN-treated mice by spectrophotometric methods. Data were presented as mean ± SD (*n* = 5/group). NS represents no significant difference.

Given *in vitro* inhibitory effect of SFN on thyroid cancer cell growth, we next determined the effect of SFN on the growth of xenograft thyroid tumors in nude mice. As shown in Figure 7A (upper panel), FTC133 cell-derived xenograft tumors progressively grew in the control mice, whereas tumors grew slowly in the mice treated with SFN at the indicated dose. Tumor volume was significantly lower in SFN-treated mice as compared to control mice (*P* < 0.001). However, body weight, an indicator of the health of the animals, did not show a significant difference between these two groups throughout the experiments (Figure 7A, lower panel). At the end of experiments, the tumors were isolated and weighted. As shown in Figure 7B, the mean tumor weight was significantly less in SFN-treated mice as compared with control mice (*P* < 0.01). To further confirm the regulation of major pathways by SFN, tumors tissues from mice were homogenated, lysed, and then subjected to western blotting using specific primary antibodies, including p-Akt, total-Akt (t-Akt), p-Erk, total-Erk (t-Erk), p-p38, total-p38 (t-p38) and active caspase-9 and E-cadherin. Similar to *in vitro* study, SFN inhibited the activity of PI3K/Akt pathway, and activated the MAPK/Erk and MAPK/p38 pathways (Figure 7C). As expected, SFN similarly enhanced the protein levels of the active form of caspase-9 and increased E-cadherin expression (Figure 7C).

To quantitatively evaluate the proliferation index of tumors treated with SFN, tumor sections were stained with the Ki-67 antibody. As shown in Figure 7D, treatment with SFN significantly decreased the number of Ki-67-positive cells in tumor as compared with controls (*P* < 0.05). More importantly, histopathological evidences demonstrated that hepatic tissues exhibited normal large polygonal cells with prominent round nuclei and eosinophilic cytoplasm, and few spaced hepatic sinusoids arranged in-between the hepatic cords with fine arrangement of Kupffer cells in both SFN-treated and control mice (data not shown). In addition, there was no significant difference in serum AST and ALT levels between SFN-treated and control mice (Figure 7E). These observations implicate that the indicated dose of SFN did not cause significant liver injury in mice, supporting that it may be a safe and effective agent for thyroid cancer therapy at least in xenograft tumor models.

## DISCUSSION

SFN, a dietary isothiocyanate found in broccoli and cauliflower, has been widely used for treatment of inflammatory diseases and recent studies have indicated its antitumor effects in cell lines and animals [14, 15, 20, 21]. Notably, a placebo-controlled, double-blind, randomized clinical study of sprout extracts containing either glucosinolates (principally the precursor of SFN) or isothiocyanates (principally SFN) was conducted on healthy volunteers in a previous study [22]. No significant or consistent subjective or objective abnormal events (toxicities) were observed, suggesting SFN is safe for human being. In addition, although a very recent phase II study showed that treatment of 200 μmoles/day of SFN-rich extracts did not lead to ≥ 50% PSA declines in the majority of recurrent prostate cancer patients, because of the safety of treatment and the effects on modulation of PSA doubling time, further studies, including those with higher dose, may be warranted to determine the role of SFN as a prevention agent or treatment agent [23]. These observations support SFN as a potential antitumor agent for cancer treatment. However, its antitumor effect in thyroid cancer remains largely unclear. In this study, we demonstrated that SFN markedly inhibited thyroid cancer cell growth *in vitro* and *in vivo*, and thyroid cancer cell migration and invasion.

Although the current evidences have highlighted the potent antitumor effect of SFN on thyroid cancer, the exact mechanism is still unclear. Our data showed that SFN-treated thyroid cancer cells were arrested in G2/M phase, which was accompanied by a decline in the levels of Cdk1, Cdk2, Cyclin B1 and Cdc25C and an increase in the levels of Chk1 and p21. ROS scavenger NAC efficiently abolished SFN-induced G2/M phase cell arrest and inhibited *p21* expression in thyroid cancer cells. It is well-documented that p53 is an important molecular target in cancer biology because it plays a central role in the regulation of cell cycle and apoptosis. Several studies have demonstrated that SFN induces a positive effect on p53 expression with a consequent increase in Bax and decrease in Bcl-2 [24, 25], whereas there are also some studies to show that there is no participation of p53 or that it is not required for the SFN activity [26, 27]. In this study, we did not find the effect of SFN on the expression of p53 protein in either p53 mutant-type FTC133 cells or p53 wild-type K1 cells. Collectively, these observations suggest that SFN up-regulates *p21* expression in these two cell lines by a p53-independent mechanism and SFN causes G2/M phase arrest by a ROS-dependent pathway, as supported by the previous studies that elevation of ROS in cancer cells caused accumulation of p21, an increase in expression of Chk1 and decrease in Cdc25C through different mechanisms including activation of Erk and p38 signaling pathways [19, 28, 29]. Accordingly, our data demonstrated that SFN enhanced phosphorylation of Erk and p38 in FTC133 and K1 cells, and the *elimination of ROS by* NAC efficiently inhibited phosphorylation of Erk and p38.

Several previous studies have shown that SFN induces ROS production, resulting in apoptosis in different types of cancers. Usually, this process is accompanied by the disruption of mitochondria membrane potential (MMP) [26, 30, 31]. It is well-known that apoptosis can be initiated in two ways: one is the “intrinsic pathway” mediated by the mitochondria, and the other is the “extrinsic pathway” mediated by death receptors. Mitochondria are the major generators of ATP by oxidative phosphorylation and mitochondria-mediated apoptosis occurs in response to a wide range of stimuli. Mitochondria are also the major source of ROS generation and damaged mitochondria can release more ROS. ROS can cause MMP disruption by activating mitochondrial permeability transition, and induce apoptosis by releasing apoptogenic protein such as cytochrome c to cytosol [32, 33]. In the cytosol, cytochrome c can activate caspase-9, and activated caspase-9 in turn cleaves and activates executioner caspase-3. After caspase-3 activation, some specific substrates for caspase-3 such as poly (ADP-ribose) polymerase (PARP) are cleaved, and eventually lead to apoptosis [34]. To explore the mechanism underlying induction of cell apoptosis by SFN in thyroid cancer cells, we tested the effect of SFN on intracellular ROS levels and MMP. As expected, our data demonstrated that SFN caused the activation of caspase-3 and -9 in thyroid cancer cells through inducing ROS generation accompanied by the loss of MMP, ultimately contributing to cell apoptosis. NAC efficiently abolish growth inhibitory and the disruption of MMP induced by SFN, suggesting that SFN-induced ROS production and mitochondrial dysfunction may be a major cause of cell death in thyroid cancer cells.

Given that tumor metastasis is the major cause of cancer-related death, we tested the effect of SFN on cell invasiveness in this study. Our data showed that SFN increased expression of E-cadherin, and decreased expression of Vimentin and E-cadherin transcriptional repressors Slug and Twist, contributing to inhibition of EMT process. This oncogenic process can be regulated by multiple signaling cascades among which PI3K/Akt cascade is the primary one [35–37]. It was consistent with our finding that SFN dramatically inhibited the activity of PI3K/Akt pathway in thyroid cancer cells by a ROS-dependent mechanism. As expected, our data demonstrated that NAC efficiently abolish the inhibition of migration and invasion by SFN, and induction of E-cadherin expression by SFN in thyroid cancer cells. Notably, a previous study has reported that NAC can not only remove ROS, but also inhibit the activity of mTOR pathway [38]. However, in this study, we did not find such effect, suggesting that NAC just plays a role as ROS scavenger in the present study. In addition, considering that matrix metalloproteinases (MMPs) play a critical role in tumor metastasis [39], we investigated the effect of SFN on expression of *MMP-2* and *-9* in thyroid cancer cells. Our data showed that SFN significantly inhibited expression of these two genes, suggesting that the decrease in the metastasis-associated phenotypes may be mediated at least in part by down-regulation of MMP-2 and -9.

Until now, numerous molecular mechanisms underlying antitumor activity of SFN have been proposed such as activation of pro-apoptotic genes such as caspase [40], blockage of tubulin polymerization [15], induction of oxidative stress [12, 13], activation of NF-κB, ERK and JNK signaling pathways [11, 14]. In recent years, it is well known that SFN inhibits the activity of histone deacetylase (HDAC), as a new mechanism underlying its pro-apoptotic effect [41, 42]. In the present study, we have shown that SFN inhibits thyroid cancer cell proliferation, migration and invasion, and induces cell cycle arrest and apoptosis through a ROS-dependent pathway. Furthermore, SFN may have a particularly high clinical potential for thyroid cancer treatment, as supported by its profound inhibitory effect on the proliferation of primary thyroid cancer cells and the growth of xenograft thyroid tumors without significant liver injury, implicating that SFN is a safe and effective antitumor agent for thyroid cancer.

## MATERIALS AND METHODS

### Human thyroid cancer cell lines and primary thyroid cancer cells

Human thyroid cancer cell lines 8305C, IHH4, FTC133, BCPAP, TPC1 and K1 were from Dr. Haixia Guan (The First Affiliated Hospital of China Medical University, Shenyang, P.R. China). C643 was from Dr. Lei Ye (Ruijin Hospital, Shanghai, P. R. China). BCPAP, TPC-1 and K1 were derived from PTC. FTC133 was derived from FTC. 8305C, C643 and IHH4 were derived from ATC. These cell lines were all routinely cultured at 37°C in RPMI 1640 medium with 10% fetal bovine serum (FBS), except for FTC133 that was cultured in DMEM/Ham's F-12 medium (Invitrogen Technologies, Inc., CA). For human primary thyroid cancer cell culturing, tumor tissues were dissected from thyroidectomy specimens. None of the patients had received prior chemical, hormonal, or radiation therapy. Histological assessment was performed by an experienced pathologist based on World Health Organization (WHO) criteria. The third passages were used for primary cell growth assays. Culture medium was DMEM/Ham's F-12 medium (Gibco-BRL, Life Technologies, NY) supplemented with 1IU/L bovine TSH (Sigma-Aldrich, Inc., MO), 10 mg/L bovine insulin (Sigma-Aldrich, Inc., MO), and 5mg/L human transferrin (Gibco-BRL, Life Technologies, NY).

In some experiments, cells were treated with sulforaphane (SFN) and ROS scavenger, N-acetylcysteine (NAC) or ascorbate (ASC) each alone or in combination as the indicated concentrations and time, and medium and agent were replenished every 24 h. SFN was purchased from LKT Laboratories. Inc. (St. Paul). NAC and ASC were purchased from Sigma-Aldrich Inc. (Saint Louis, MO), and dissolved in dimethylsulfoxide (DMSO) and Tris-HCl (pH 7.4), respectively, aliquoted and stored at −80°C until use. The same volume of vehicle was used as control.

### Cell proliferation assay

Cells (3000/well) were seeded into 96-well plates and cultured with different concentrations of SFN for 72 h. 3-(4, 5-Dimethylthiazolyl-2)-2, 5-diphenyltetrazolium bromide (MTT) assay was used to evaluate cell proliferation. IC_50_ values were calculated by using the Reed-Muench method [43]. After SFN treatment at the indicated times, cell culture was added with 10 μl of 5 mg/ml MTT (Sigma, Saint Louis, MO) and incubated for 4 h, followed by addition of 150 μl of DMSO for 15-min. The plates were then read on a microplate reader using a test wavelength of 570 nm and a reference wavelength of 670 nm. Three triplicates were done to determine each data point.

### Cell cycle analysis

Cells were serum starved for 12 h. After co-culture with the indicated doses of SFN and/or NAC for 24 h, cells were harvested, washed twice in PBS, and fixed in 70% ethanol on ice for at least 30 min. Cells were then stained with propidium iodide (PI) solution (50 μg/ml PI, 50 μg/ml RNase A, 0.1% Triton-X, 0.1mM EDTA). Cell cycles were analyzed based on DNA contents using a flow cytometer (BD Biosciences, NJ).

### Apoptosis assay

Cells (4 × 10^5^/well) were plated into 6-well plates. After 24 h-incubation, cells were treated with the indicated concentrations of SFN and/or NAC. After 12 h-, 24 h- and 36 h-incubation, cells were harvested, washed with PBS, and subjected to sequential staining with Annexin V-FITC/PI Apoptosis Detection Kit (Roche Applied Science) by two-color flow cytometer (BD Biosciences, NJ), according to the manufacturer's protocol. Each experiment was performed in triplicate.

### RNA extraction and real-time quantitative RT-PCR (RT-qPCR) analysis

Total RNA was isolated from cells using TRIzol (Takara Inc., Dalian, P.R. China) according to the instructions of the manufacturer. Total RNA (500 ng) was converted to cDNA using PrimeScript RT reagent Kit (Takara Inc., Dalian, P.R. China) according to the instructions of the manufacturer. RT-qPCR was performed to evaluate expression of the indicated genes on a CFX96 real-time PCR system (Bio-Rad Laboratories, Inc., CA), using SYBR Premix Ex*Taq* II (Takara Inc., Dalian, P.R. China) with *18S* rRNA cDNA as endogenous control. The primer sequences are presented in Supplementary Table S1. Each sample was run in triplicate.

### Western blot analysis

Cells were lysed in RIPA buffer containing protease inhibitors. Cellular proteins were collected and subjected to 10% SDS-PAGE, and transferred onto PVDF membranes (Roche Diagnostics, Mannheim, Germany). The membranes were then incubated with the indicated primary antibodies. Anti-caspase-3 (H-277) and caspase-9 (96.1.23) were purchased from Santa Cruz. Anti-E-cadherin and anti-Vimentin were purchased from Epitomics, Inc. Anti-phospho-Akt^Ser473^ (p-Akt), anti-total-Akt (t-Akt), anti-phospho-Erk (p-Erk) and anti-total-Erk (t-Erk) were purchased from Bioworld Technology. Anti-phospho-p38 (p-p38) and anti-total-p38 (t-p38) were purchased from Cell Signaling Technology. Anti-JNK1+JNK2+ JNK3 (phospho T183+T183+T221) antibody was purchased from Abcam. Anti-p53 was purchased from ZSGB-Bio. Anti-GAPDH was purchased from Abmart. This was followed by incubation with species-specific HRP-conjugated secondary antibodies from ZSGB-BIO, and antigen-antibody complexes were visualized using the Western Bright ECL detection system (Advansta, CA).

### Detection of reactive oxygen species (ROS) and mitochondrial membrane potential (MMP)

Generation of ROS was measured by the oxidation-sensitive fluorescent probe Dichloro-dihydro-fluorescein diacetate (DCFH-DA) as previously described [44]. Briefly, 2 × 10^6^ cells were treated with 40 μM SFN and/or 20 mM NAC for 4 h. Cells were then harvested, washed, and suspended in serum-free medium containing 10 μM of DCFH-DA at 37°C for 30 min in the dark. Cells were subsequently washed and resuspended in PBS, and analyzed by flow cytometer.

JC-1 is a kind of ideal fluorescent probe widely used in the detection of mitochondrial membrane potential (MMP). Following cells treated with 40 μM SFN and/or 20 mM NAC for 4 h, JC-1 were added to the culture medium (500 μl/well) and incubated at 37°C for 30 min in the dark for mitochondrial staining. After washing twice with cold dyeing buffer to remove unbound dye, resuspended in dyeing buffer and inspected by fluorescence microscopy. Quantification by flow cytometry detected mitochondria containing red JC-1 aggregates in the FL2 channel and green JC-1 monomers in the FL1 channel.

### Cell migration and invasion assays

Cell migration and invasion assay was performed using Transwell chambers (8.0 μm pore size; Millipore, MA). It was noted that, for cell invasion assay, the chambers were coated with 24 μg/μl of Matrigel (BD Bioscience, NJ) in 24-well plates. Cells (1 × 10^4^/well) were seeded in the upper chamber in 400 μl of medium containing 0.5% FBS and the indicated concentrations of SFN or vehicle control. Medium with 10% FBS (600 μl) was added to the lower chamber. Following a 24 h-incubation at 37°C with 5% CO_2_, non-migrating and non-invading cells in the upper chamber were removed with a cotton swab, migrating and invading cells were fixed in 100% methanol and stained with 0.5% crystal violet in 2% ethanol. Photographs were taken randomly for at least five fields of each membrane using a microscope. The number of migrating or invading cells was expressed as the average number of cells per microscopic field over five fields. All assays were run in triplicate.

### Xenograft tumor assay in nude mice

Female athymic nude mice were purchased from SLAC laboratory Animal Co., Ltd. (Shanghai, PR. China) and housed in a specific pathogen-free (SPF) environment. Thyroid cancer cells FTC133 (3 × 10^6^) were injected s.c. into flanks of mice at the age of 5 wk. When tumors grew to ∼5 mm in diameter, mice were grouped into two groups (five mice per group). Two groups were treated with vehicle control or 50 mg/kg SFN through i.p. injection every 2 days, respectively. Tumor volumes were measured at the start of the treatment every 2 days during the course of the therapy. Tumor volumes were calculated by the formula (width)^2^ × length/2. After treatment for 13 days, tumors were harvested and weighted.

Tumors tissues from representative mice were homogenated and lysed for western blotting analysis. The remaining tissues were embedded in paraffin, sectioned at 4 μm, and stained with hematoxylin and eosin (H&E). Cell proliferation ability was assessed by quantification with Ki-67 immunohistochemistry. Briefly, anti-human Ki-67 antibody (BD Pharmingen) was 1:150 diluted and immunostaining was done according to a standard protocol using DAB Substrate Kit (ZSGB-BIO). Ki-67-positive cells were scored by visual examination of 5 randomly selected fields of ×200 magnification containing at least 1000 cells. Moreover, to evaluate the effect of SFN on hepatocyte damage of animals, we performed H&E staining of liver sections and detected serum activities of aspartate transaminase (AST) and alanine transaminase (ALT) by spectrophotometric methods as previously described [45].

### Statistical analysis

Data were compared using the *t* test (SPSS statistical package 16.0, Chicago, IL). A *P* < 0.05 was considered to be statistically significant. All values were expressed as the mean ± SD. Unless indicated, data shown in the figures are representatives.

## SUPPLEMENTARY FIGURES AND TABLES


