# Supplementary material for: Sulforaphane inhibits thyroid cancer cell growth and invasiveness through the reactive oxygen species-dependent pathway
Source: Oncotarget. 2015 Jul 17;6(28):25917–31. doi: 10.18632/oncotarget.4542 (PMC4694875; doi:10.18632/oncotarget.4542)
Supplement: Supplementary file 1 [file oncotarget-06-25917-s001.pdf]

SUPPLEMENTARY FIGURES AND TABLE

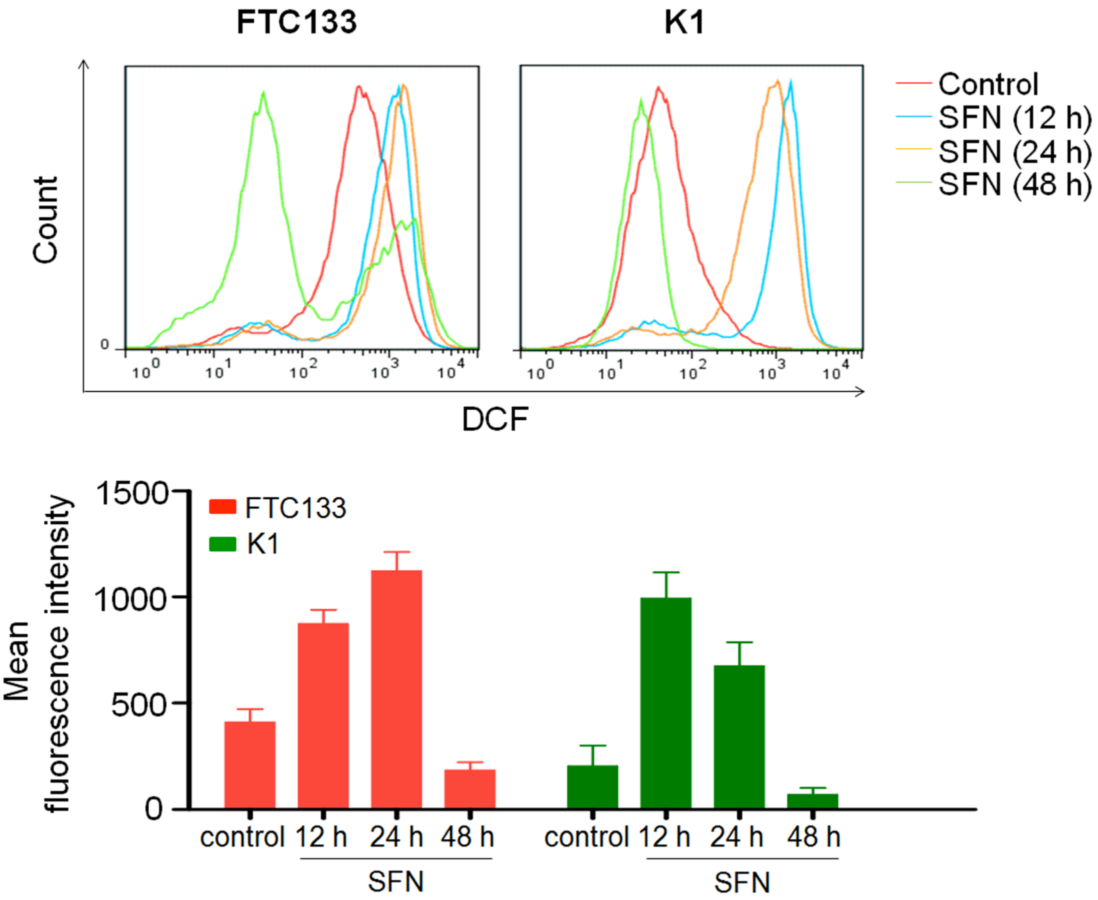

Supplementary Figure S1: The effect of SFN on ROS production. FTC133 and K1 cells were treated with 40 µM SFN for 12 h, 24 h and 48 h. DCF staining was used to evaluate ROS production. The fluorescence intensity of ROS-positive cells is shown in the upper panel. The lower panel shows mean ± SD of the values from three independent experiments in these two cell lines.

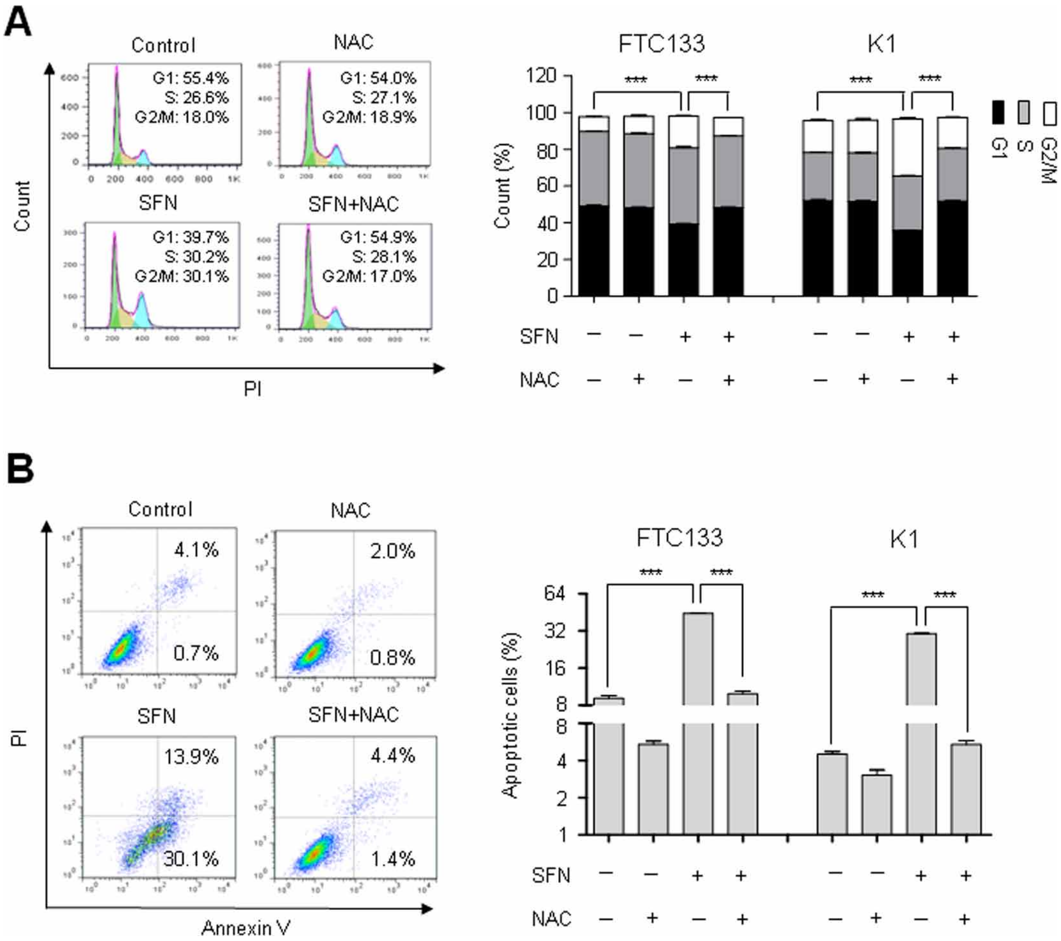

Supplementary Figure S2: Effect of ROS on cell cycle arrest and apoptosis induced by SFN. A. DNA content was measured by flow cytometry to determine cell cycle fractions. Representative flow cytometric histograms of FTC133 cells treated with 20 μM SFN and 20 mM NAC each alone or in combination for 24 h (left panel). The distribution and percentage of FTC133 and K1 cells in each cell cycle phase is indicated in the right bar graph. ***, $P < 0.001$. **B.** Cell apoptosis was measured by flow cytometry analysis of Annexin V-FITC/PI double-labeled cells treated with 20 μM SFN and 20 mM NAC each alone or in combination for 24 h. Flow cytometry profile represents FTC133 cells with Annexin V-FITC staining in *x*-axis and PI in *y*-axis (left panel). Dual staining of cells with Annexin V-FITC and PI enabled categorization of cells into four regions. Upper right region shows late apoptotic cells, lower right region shows early apoptotic cells. These two regions are collectively called apoptotic cells. The percentage of apoptotic cells for FTC133 and K1 cells is indicated in the right bar graph. ***, $P < 0.001$.

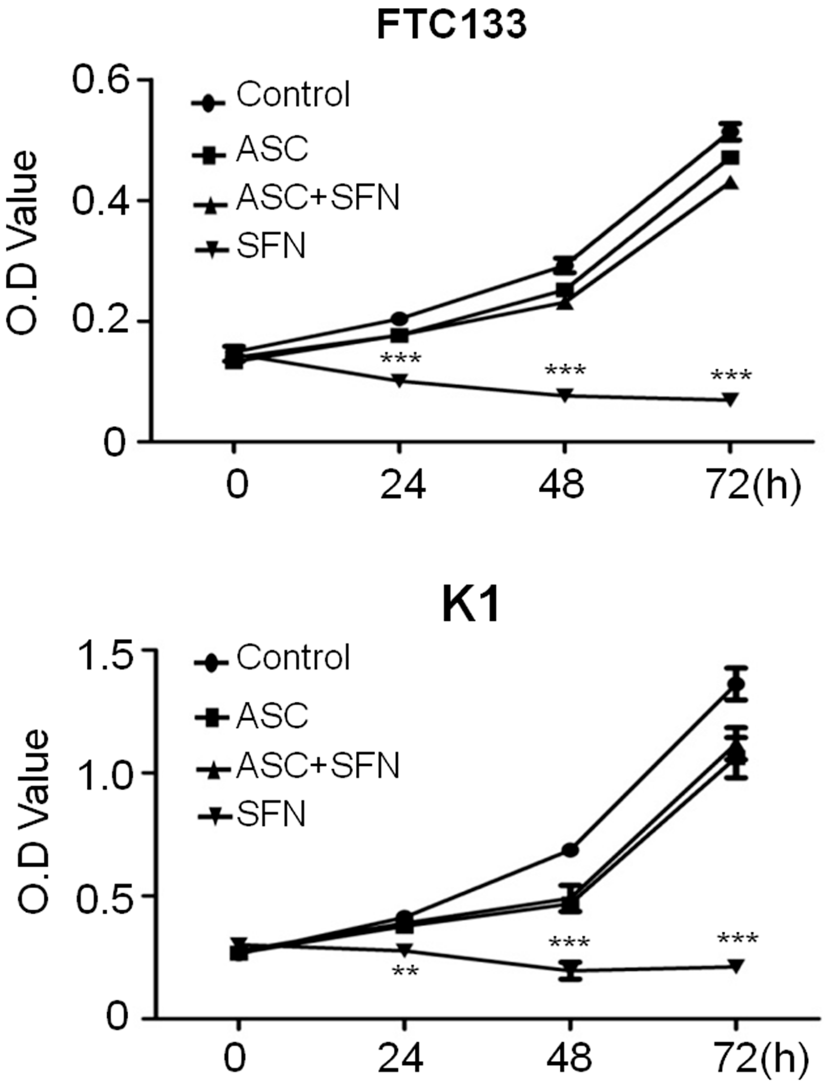

Supplementary Figure S3: The effect of elimination of ROS by ASC on thyroid cancer cell proliferation. Time course of cell proliferation was measured by MTT assay in FTC133 and K1 cells treated with 40 µM SFN and 1 mM ASC each alone or in combination. **, $P < 0.01$; ***, $P < 0.001$.

Supplementary Table S1: RT-qPCR primers used in this study

Gene	Forward primer (5′-3′)	Reverse primer (5′-3′)	Product length (bp)
Cdk1	TTGGATTCTATCCCTCCTGG	CTGGAGTTGAGTAACGAGCTGA	283
Cdk2	TGGTACCGAGCTCCTGAAAT	GAATCTCCAGGGAATAGGGC	122
Cdc25C	CCTAGGAGAAGACCAGGCAG	GCCTGTTCAAGTTCTCTGGC	128
Chk1	TGGGCTATCAATGGAAGAAAA	TCATCCATTTCTAACAAATTCACTT	106
CyclinB1	GAACCTGAGCCAGAACCTGA	ACAGGTCTTCTTCTGCAGGG	124
MMP-2	GGAAAGCCAGGATCCATTTT	ATGCCGCCTTTAACTGGAG	103
MMP-9	TTGGTCCACCTGGTTCAACT	ACGACGTCTTCCAGTACCGA	95
p21	ACCTGTCACTGTCTTGTACC	GTAGAAATCTGTCATGCTGGTC	119
Slug	TGACCTGTCTGCAAATGCTC	CAGACCCTGGTTGCTTCAA	94
Twist	GTCCGCAGTCTTACGAGGAG	GTCTGAATCTTGCTCAGCTTGTC	148
Vimentin	GGACCAGCTAACCAACGACA	AAGGTCAAGACGTGCCAGAG	178
18S	CGCCGCTAGAGGTGAAATTC	CTTTCGCTCTGGTCCGTCTT	52
